# DOES PERCEIVED EFFICACY OF COGNITIVE TRAINING BIAS OUTCOMES?

**DOI:** 10.1093/geroni/igac059.2081

**Published:** 2022-12-20

**Authors:** Shenghao Zhang, Nicholas Gray, Neil Charness, Walter Boot

**Affiliations:** Florida State University, Tallahassee, Florida, United States; Florida State University, Tallahassee, Florida, United States; Florida State University, Tallahassee, Florida, United States; Florida State University, Tallahassee, Florida, United States

## Abstract

Cognitive training has the potential to help older adults maintain or improve their cognitive abilities, but there has been debate regarding how susceptible cognitive outcomes in these studies are to placebo effects. Some previous research has found that expectations can shape performance on study outcomes, although this is not always a consistent finding. The degree to which perceived intervention efficacy can lead to changes in the performance of objective outcome measures typically assessed in these studies is unclear. The aim of the current study is to explore the effects of perceived effectiveness of cognitive training on actual improvements in a large sample of older adults. The current study utilized data from the Intervention Comparative Effectiveness for Adult Cognitive Training (ICE-ACT, NCT03141281) Trial. Two hundred and thirty older adults (Mean age = 72) were randomized into one of four training conditions: (1) a web-based brain game suite, Brain HQ, (2) a strategy video game, Rise of Nations, (3) Instrumental Activities of Daily Living (IADL) training, or (4) an active control condition of puzzle solving. Reasoning, memory, processing speed, and IADL performance were measured before and after the intervention. Perceived efficacy of training in those outcome domains were also measured. Bayesian ANOVA analysis showed strong evidence of group differences in perceived efficacy within all outcome domains across the training conditions whereas Bayesian regression showed moderate to strong evidence against influences of perceived efficacy on changes in performance. Discussion will focus on implications of those results on behavioral and cognitive intervention designs.

